# Effects of Health-Related Behaviors and Changes on Successful Aging among Indonesian Older People

**DOI:** 10.3390/ijerph19105952

**Published:** 2022-05-13

**Authors:** Lisa Wahidatul Oktaviani, Hui-Chuan Hsu, Yi-Chun Chen

**Affiliations:** 1 School of Public Health, Taipei Medical University, Taipei 11031, Taiwan; lwo827@umkt.ac.id; 2 Department of Public Health, Universitas Muhammadiyah Kalimantan Timur, Samarinda 75124, Indonesia; 3 Research Center of Health Equity, College of Public Health, Taipei Medical University, Taipei 11031, Taiwan; yichun@tmu.edu.tw; 4 School of Nutrition and Health Science, Taipei Medical University, Taipei 11031, Taiwan

**Keywords:** health behaviors, physical activity, smoking, protein intake, older people, successful aging

## Abstract

Whether changes in health behaviors can improve successful aging has not been well explored. The purpose of this study was to assess the effects of health-related behaviors and changes on successful aging in Indonesian older adults. Data were from the fourth and fifth waves of the Indonesia Family Life Survey (IFLS), the participants were aged 60 years and older and who completed both waves (*n* = 1289). Successful aging indicators were defined as no chronic diseases, no physical function difficulties, no depressive symptoms, intact cognitive function, with social support, and with social participation. Health-related behaviors focused on smoking, physical activities, and protein intake. A logistic regression analysis was conducted. The overall successful aging rate in 2007 was 23.6%, and it had decreased to 5.6% by 2014. There were gender differences in smoking, physical activities, and behavioral changes, including promoting increased physical activity, no smoking/smoking cessation, and adequate protein intake by older adults. Quitting smoking, performing medium physical activity, and increasing protein intake were protective factors for successful aging, but the effects of behavioral changes differed by gender. Health-related behaviors and changes may impact successful aging among older adults. A healthy lifestyle is suggested to be adopted as early as possible in one’s life course.

## 1. Introduction

The concept of successful aging has become the new paradigm in gerontological research. According to the most widely applied definition by Row and Khan [1], successful aging is the status of older people simultaneously meeting the following criteria: a low risk of diseases, high cognitive and physical functioning, and engagement with life. The concept of successful aging has been explored or applied in Western countries for a long time [2,3,4,5]. Similar studies were conducted in Asian countries as well, including Taiwan [6,7], Singapore [8], Korea [9], Japan, China [10], and Indonesia [11]. Early studies focused on how to define successful aging, and later studies explored factors related to successful aging by applying longitudinal data. Although associations of health-related behaviors with successful aging were also explored [12], the effects of changes in health behaviors on successful aging have been less well explored. In particular, due to differences in social roles by gender, health behaviors and successful aging may differ by gender. In this study, we examined the successful aging status of older people in Indonesia and the effects of changes in healthy behaviors on successful aging.

### 1.1. Heath-Related Behaviors and Successful Aging

A successful aging process is not only impacted by implementation of public policies that promote healthy aging but also depends on outcomes of individual behaviors, i.e., behaviors that avoid risk factors and strengthen protections throughout one’s life [13]. Physical activities, smoking, and dietary patterns have all been linked to components that are frequently included in successful aging criteria [14]. Older adults engage in healthy behaviors, including smoking cessation [15,16,17], having good dietary protein intake [18,19,20], and performing physical activities [16,21,22,23,24,25]. Certain behaviors may contribute to successful aging by several previous longitudinal studies, such as smoking cessation, which affect the absence of disease [12], physical functioning [12,26], cognitive functioning [15,27], and active engagement with life [12]. Engaging in physical activities is beneficial for survival, physical functioning [12,28], and cognitive functioning [15]. Reduced salt intake and saturated fat intake may reduce the morbidity due to chronic heart diseases [29]. A sedentary lifestyle is related to greater depressive symptoms [30]. Protein intake increases muscle mass and physical functioning [19,25,31]. Alcohol consumption may be related to declines in physical functioning [12] and cognitive functioning [15]. An integrated healthy lifestyle is related to a better chance of overall successful aging [11,14].

### 1.2. Changes in Health-Related Behaviors and Successful Aging

Adopting a healthier lifestyle by changing behaviors is beneficial not only for individuals who have health problems in their old age but also for healthcare professionals and the government to maintain their healthcare and social care budgets [32]. Some of the effects of changes in health behaviors on successful aging were illustrated by several previous studies. Understanding food-intake patterns of older people is a strategy to overcome malnutrition in this population. Older adults are advised to increase their protein intake compared to younger adults to maintain health, promote disease recovery, and maintain function [33]. Increased protein intake may delay the incidence of frailty in very old adults [34]. Changes in physical activity among older adults are associated with successful aging [16,30,35,36,37]. Higher physical activity is associated with better mental health and the prevention or reduction of depressive symptoms [30,35,36]. Changing sedentary habits into light physical activity is associated with improving health [37] and cognitive functioning [16]. Older smokers have higher risks to cognitive functioning [16,17] and higher morbidity and mortality [38], while reducing smoking may cause depression [39,40] yet boost overall health [41]. Changes in the smoking status were associated with successful aging as well. Consistently smokers showed significant cognitive declines compared to non-smokers [16]. Quitters and non-smokers have decreased risks of dementia [42], and smoking is related to faster declines in cognitive function [15]. A meta-analysis showed that smoking cessation was related to a lower possibility of depression compared to current smokers [43], and smoking cessation may reduce the risk of frailty [44]. There was a dose-response relationship of smoking with the risk of mortality [45], and reducing the number of cigarettes smoked per day reduces the risk of mortality [46]. Changing sedentary habits into light physical activity associated with improving the health [37] and cognitive function [16]. Alcohol drinking was found to be related to successful aging in longitudinal studies [12,15], but the effects of the change of alcohol intake among older adults were less explored.

### 1.3. Gender Differences in Health-Related Behaviors and Successful Aging

Gender differences in several dimensions of successful aging were found. Older men are more likely to have a better status of successful aging. Gender differences are associated with multimorbidity [47,48], physical functioning [49,50,51], cognitive functioning [52,53,54], depression [55,56,57], and engagement with life [58,59,60]. Gender differences were also found in health-related behaviors, including physical activity levels [61,62], smoking [63,64], and protein intake [65,66,67,68,69]. Older men are more likely to smoke, do more physical activities, and have higher protein intake than older women. Gender differences in health and use of health behaviors can be explained as different social classes between men and women and show social norms. Women tend to have lower social positions than men in most societies. Social position mediates access to positive and negative social and environmental factors that occur in the individual, household, and community levels [70,71]. Due to the cumulative disadvantage, women usually have fewer chances to get education and promotion in their jobs, which contributes to a lower income [72]. Gender differences in HRBs can also be explained by the knowledge gap or health literacy differences, differences in health beliefs, social role differences, and social disparities due to gender [73].

### 1.4. Background in Indonesia

In Indonesia, in almost five decades (1971~2020), the percentage of elderly has more than doubled to 9.92% (about 26 million people) [73]. The rise in the number of older people coincides with an increase in the weight of dependency on family, society, and the government. However, the successful aging status has been little explored in Indonesia. In particular, high smoking rate in males, low physical activity in females, and popular consumption of fried food are major health issues in Indonesia [74]. The Ministry of Health of Indonesia has started health promotion for older people through the Posyandu Lansia program to provide physical and mental health checkups and monitor health behaviors (including smoking history, dietary history, and physical activity) for older people. Nevertheless, the effects of smoking, low physical activity, and dietary patterns on successful aging for older people in Indonesia have not been explored.

Many studies have used longitudinal data to examine factors related to successful aging [7,15,16,20,38,39,40,41,42,45,46,59,63,68], and changes in health behaviors may affect health outcomes [12,15,16,30,31,32,34,35,36,37,38,39,40,42,44,45,46]. However, whether changes in health behaviors affect the chance of successful aging for older adults in Indonesia has not been confirmed. In addition, whether gender differences in health behaviors cause differences in successful aging has not been examined either. Thus, the purposes of this study were to examine related factors and the effects of changes in health-related behaviors on successful aging by older people in Indonesia using longitudinal data.

In this study, we have not just used a longitudinal data but also have examined the changes of health-related behaviors and particularly focused on the effects of changes of behaviors on successful aging. The findings are expected to provide suggestions for health-promoting policies and implications for further research related to older people.

## 2. Materials and Methods

### 2.1. Data and Sample

A longitudinal study design was used in this study to examine associations of related factors at the baseline with successful aging among older Indonesian adults. Data were obtained from the Indonesia Family Life Survey (IFLS), a longitudinal socioeconomic and health survey. It is based on a representative sample of Indonesian families. The current study used the fourth and fifth waves of the IFLS, which were conducted starting from 2007 and 2014, respectively. The target population in this study included those aged ≥ 60 years in 2007 (wave 4) and who completed the follow-up in 2014 (wave 5). In total, the sample we analyzed consisted of 1289 individuals.

### 2.2. Measures

Successful aging indicators in wave 5 were the dependent variables, and independent variables in wave 4 and changes in those variables between waves 4 and 5 were included in the model.

#### 2.2.1. Successful Aging

Successful aging was defined according to Rowe and Kahn’s framework [1]. In this study, successful aging was measured as participants who simultaneously met six indicators in three domains: physical (free of chronic disease and no difficulties with instrumental activities of daily living (IADLs), psychological (no depressive symptoms and intact cognitive function), and social (having good social support and social participation).

1. No chronic diseases: Nine chronic diseases were assessed by self-reporting, including hypertension, diabetes, asthma, heart attack, liver, stroke, cancer, arthritis, and gout. The variable was coded as 0 (having disease) or 1 (not having any disease).
2. No physical function difficulties: IADLs were used to determine physical functioning. IADL items included shopping for personal needs, preparing food, and taking medications. Each item was coded 0 (easy), 1 (somewhat difficult), and 2 (unable to do) and then summed. The total score ranged 0–6 and was then recoded as 0 (having any difficulty) or 1 (having no difficulty).
3. No depressive symptoms: The Center of Epidemiological Studies Depression Scale (CESD)-10 was used to measure depressive symptoms. Each item was scored from 0 to 3, and the total score ranged 0~30. A total score of >10 was defined as having depressive symptoms (yes = 1; no = 0) [75].
4. Intact cognitive function: Cognitive function was measured by the Telephone Survey of Cognitive Status (TICS) [76] using the following assessments: (1) awareness of the date (scored 0~2); (2) awareness of the day of the week (scored 0~1); (3) word recall of 10 nouns (scored 0~9); and (4) second time to repeat 10 nouns (scored 0~9). A score of ≤6 was indicative of impaired cognitive function, and a score more than 6 was defined as intact (1 = intact; 0 = impaired).
5. Having social support: Living with spouse and children (yes = 1; no = 0).
6. Having social participation: Social participation was defined as involvement in five types of community groups or activities in the previous 12 months (yes/no): community meetings, volunteer labor, programs to improve the neighborhood, religious activities, and Arisans. An Arisan is a group of people who contribute money on a regular basis over a set period of time. After the money has been raised, one of the members will be proclaimed the winner, and the winner will be responsible for holding the next meeting. The Arisan helps people save money, build friendships, and increase social interactions [77]. Social participation was described as those who participating in at least one type of community activity. The coding of social participation was defined as yes (1) or no (0).

The six indicators above were used to describe successful aging. Participants who had no chronic diseases, had no physical function problems, had no depressive symptoms, had no diminished cognitive function, had social support, and participated in social activities were considered to be successfully aging. In addition, the three domains of successful aging were also defined for analysis: physical successful aging (no chronic diseases and no physical function difficulties), psychological successful aging (not depressive and without impaired cognitive function), and social successful aging (having good social support and social participation).

#### 2.2.2. Related Factors

Related factors included demographics and health-related behaviors at the baseline and changes that occurred between the two waves. Demographic variables included age (60~69 and ≥70 years), gender (male and female), educational level (college, university and above, senior high school, junior high school, elementary, and no formal education), monthly expenditure (USD 2.98~26.91, USD 26.92~39.62, USD 39.63~56.50, USD 56.51~91.45, and ≥USD 91.43), place of residence (rural and urban), ethnicity (Javanese and non-Javanese ethnic groups), and health insurance (yes and no).

Three health-related behaviors were assessed: smoking, physical activities, and protein intake. Smoking was classified as yes (1 = current smoker) or no (0 = non-smoker). The brief version of the International Physical Activity Questionnaire (IPAQ) [78] was used to assess physical activity by asking about the number of days expended on three tasks in the previous 7 days: vigorous activities, moderate physical effort, and walking. Physical activity was divided into three categories based on the criteria of the brief IPAQ: high (at least 3 days in a week of vigorous-intensity activity), moderate (at least 5 days of moderate-intensity activities and/or 30 min of walking per day), and low (not meeting any criteria for vigorous or moderate exercise). The coding was physical activity was low (0), moderate (1), and high (2). Protein intake was assessed using the results of a food intake questionnaire. The Food Frequency Questionnaire (FFQ), a standard instrument for measuring food intake, was used to determine dietary intake [79]. Protein food items were inconsistent in the two waves, and thus, only consistent food items were included in the analysis. We used four food items: eggs, fish, meat (beef, chicken, pork, etc.), and dairy products. Protein intake was assessed as high protein intake (intake at least once every day of any kind) and low intake (not every day) (high = 1, low = 0). Changes in certain variables in the two waves were included in the analysis: place of residence, health insurance status, smoking, protein intake, and physical activity.

### 2.3. Analysis

Descriptive analyses, bivariate analyses, and binary logistic regression analyses were used in this study. Changes in factors related to successful aging were analyzed by a binary logistic regression.

## 3. Results

Table 1 shows the characteristics of the sample population. In the two waves, the smoking rate dropped from 55.1% to 47.6%. There were 56.6% of older participants with low physical activity, 18.9% had medium, and 24.5% had high physical activity levels at the baseline; physical activity slightly increased in the follow-up wave. There were 61.8% of participants with high protein intake at the baseline, but only 53.7% had high protein intake in the follow-up. Over a span of 7 years, the proportion of the six indicators of successful aging decreased from the baseline.

Changes in health behaviors by sex are shown in Table 2. Most of the participants maintained stable health behaviors, but there were gender differences in health behaviors. There were gender differences in smoking: most males continued to smoke (71.2%), while most females maintained a non-smoking status (79.5%). The starting smoking rate and quitting rate were slightly higher in males than in females. Regarding changes in the pattern of protein intake, 23.9% maintained a low status, 39.5% maintained a high status, 14.3% increased their protein intake, and 22.3% reduced their protein intake. There were no gender differences in changes in protein intake patterns. As for changes in physical activity, 40.5% remained stable, while 26.2% reduced their physical activity, and 33.3% increased it. Gender differences were found in physical activity changes with older females having a higher stable rate (44.1%) than males (36.6%) and with older males showing greater increases in physical activity (37.2%) than older females (29.7%).

Table 3 shows the successful aging rate by gender. In 2007, older males showed significantly better successful aging rate than older females in no chronic diseases, no cognitive impairment, having social support, having social participation, and overall successful aging. In 2014, older men still showed better successful aging in most of the indicators except there was no difference in depressive symptoms. That indicates the older men had a better chance at successful aging than older women. The bi-variate analysis of the association of successful aging with baseline and the changes of the independent variables by gender are shown in the Supplementary Materials Tables S1 and S2. There were gender differences in health behaviors and successful aging. Thus, we analyzed the association of health-related behaviors with successful aging by gender in the following analysis.

Longitudinal changes in health-related behaviors and factors related to successful aging among older males by the logistic regression are presented in Table 4. Older adults who had no chronic diseases were more likely to have low education (odds ratio (OR) = 0.75), low monthly expenditures (OR = 0.85), a stable health insurance status between the two waves (OR = 0.56 from no to yes), performed medium physical activity at the baseline (OR = 1.83), and had a change in the smoking status (OR = 0.41 for quitting smoking). Those with no difficulties in physical functioning were more likely to perform low physical activity (OR = 0.56 for moderate physical activity) and to have experienced a protein intake change from low to high (OR = 2.18). Those who had no cognitive impairment were more likely to be younger (OR = 0.25 for older) and have higher education (OR = 1.97). Those with greater social support were more likely to have stayed and not changed their residence between the two waves (OR = 0.46 to change one’s residence), to have changed their health insurance status from not having to having insurance (OR = 2.26), and to have changed their smoking behavior from having smoked at the baseline (OR = 0.21) to having quit smoking (OR = 0.22). Older adults who had good social participation were more likely to be younger (OR = 0.55 for older), have a higher educational level (OR = 1.44), be Javanese (OR = 1.95), and have increased their physical activity levels between the two waves (OR = 1.98). Overall successful aging, as assessed by meeting all six indicator criteria, was significantly related to being younger (OR = 0.21 for older).

Health-related behavioral changes and factors related to longitudinal successful aging among older females according to the logistic regression are presented in Table 5. Older adults who had no chronic diseases were more likely to have lower monthly expenditures (OR = 0.85) and to have changed their health insurance status from yes to no (OR = 2.02). Those with no physical function difficulties were more likely to have low changes in stable protein intake between the two waves (OR = 0.50 for a low to high change). Those who had no cognitive impairment were more likely to be younger (OR = 0.33 for older), have a higher educational level (OR = 2.52), and have increased performance of physical activities (OR = 1.75) between the two waves. Those with good social support were more likely to be younger (OR = 0.33 for older) and Javanese (OR = 1.70). Older adults who had good social participation were more likely to have a higher educational level (1.72), be Javanese (OR = 2.23), and have increased their level of physical activity (OR = 1.79) between the two waves. Those with overall successful aging, as assessed by meeting all six indicator criteria, were more likely to have had a higher educational level (OR = 2.24) and to have changed their residence (OR = 4.51).

Furthermore, we tried to add age interaction terms with health behavioral changes and education interactions with behavioral changes to examine the possible moderating effects of age and socioeconomic status in health behavior changes. Please see the results in the Supplementary Materials (Tables S3 and S4). Most of the age interactions and education interactions were not significant and may dilute or offset the original age and education effects; an exception is higher education with high protein intake, which may be protective in cognitive function for males and facilitate overall successful aging for females.

## 4. Discussion

In this study, we examined effects of related factors and changes in health-related behaviors on successful aging by examining longitudinal data of older people in Indonesia. The overall successful aging rate in 2007 was 23.6%, which had decreased to 5.6% by 2014 based on the six indicators. There were significant gender differences in smoking, changes in smoking, and changes in physical activity as well as successful aging indicators. For older males, smoking was related to a lower chance of having social support, and those quitting smoking also had lower chances of having no chronic diseases and having social support. Performing a medium level of physical activity was related to a better chance of having no chronic diseases but a smaller chance of having no physical difficulties, while increased physical activity increased the chance of having social participation. Males with a change in protein intake from low to high had an increased likelihood of having no physical functional difficulties compared with those who maintained a low protein intake. Regarding older female participants, physical activity at the baseline was not significant, but higher physical activity increased the chances of intact cognitive functioning and having social participation. Changing one’s protein intake from low to high was related to a lower chance of having no physical functional difficulties compared to the group who maintained a low protein intake.

### 4.1. Successful Aging in Indonesia

We compared the successful aging rates of Indonesian older adults with those in China, Japan, Singapore, and South Korea, which were assessed at about the same time [8,9,10], although the measurements were not exactly the same. The success rate in Korea was 13.3% [9], and those in China and Japan were 15.7% and 29.2%, respectively [10]. Successful aging rates of Indonesia were higher in 2007 (23.6%) and decreased to much lower in 2014 (5.6%) compared with these countries.

The successful rate of these six indicators of Indonesia with these Asian countries are also compared. The rate of having no chronic diseases in Indonesia was higher than those in Korea (48.2%) and China (40.4%) and was only lower than the early developed country of Japan (62.1%). The low rate of chronic diseases in Indonesia is due to the fact that most people do not realize they have a chronic disease and only seek health services when they have had an acute attack or have a disability due to their illness. Indonesia just recently initiated a universal health insurance scheme in 2014. There may have been unmet needs of healthcare utilization before implementation of universal health insurance, and thus, the self-reported morbidity may have been underestimated. That implies the measurement of successful aging from Rowe and Kahn’s model, at least at the current stage, might not be a perfect indicator for older people in Indonesia.

Indonesia also had the highest rate for active engagement compared to China, Korea, and Japan, especially in terms of social participation. In Indonesian culture, people are expected to be friendly and easy to get along with in their neighborhood, particularly in rural areas. Older people in Indonesia often participate in different kinds of social groups, and *Arisan* and religious groups are the most popular kinds. *Arisan* groups are a unique activity with chances to save money, meet friends, and increase social interactions [77]. *Arisan* is an activity in which all age groups in Indonesia participate and demand, even many elementary school-aged children form *Arisan* groups. In addition, Indonesia requires that all people need to have a religion belief. For example, *Pengajian,* a Muslim community activity, is an arrangement of religious activities that aims to produce an experience of religious teaching. Such activities are highly demanded in the community, especially by older adults. Participating in such groups make older people feel closer to God and happier, and that also benefits higher cognitive functioning and quality of life [43]. Thus, older people tend to participate in various kinds of social groups and enjoy highly social connectedness compared to older people in other countries.

### 4.2. Smoking and Smoking Changes and Successful Aging

Most smokers were males among these older people. In this study, older males who quit smoking had a lower chance in having no chronic diseases. It is possible that older males quit smoking because they were sick from chronic diseases. We also found that older males who smoked at the baseline and subsequently quit smoking were less likely to have social support (i.e., living with family). Older men who did not have a spouse were more likely to smoke [80]. Further, many families in Indonesia find it very difficult to accept if there are family members who smoke when they are older. Smoking may lead to diseases and becomes a caregiving burden on the family.

### 4.3. Physical Activity and Changes and Successful Aging

We found that older males who performed moderate physical activity were more likely to have no chronic diseases, which is consistent with previous studies [81,82,83]. However, those who performed medium physical activity were less likely to have no physical difficulties than those who performed lower or no activities. One explanation is that some older adults did have the habit of physical activity until frailty or functional limitations occurred. For older participants in this study, performing physical activities was more like a response to functional difficulties but not a cause of physical functioning. The causal relationship between physical functional difficulties and physical activities needs to be confirmed with further information. Those who increased their physical activity over a period of 7 years were more likely to have good social participation. Many kinds of social participation require good physical functioning to participate in the activities, and increasing physical activities usually is beneficial for physical functioning. Older female participants who increased their physical activity were more likely to have intact cognitive functioning. This is in accordance with previous studies [16,84], and those with increased physical activity were also more likely to have social participation.

### 4.4. Protein Intake and Changes and Successful Aging

In this study, older males whose protein intake changed from low to high were more likely to having no physical difficulties, which is consistent with previous studies [33,34]. However, older women who experienced changes in protein intake from low to high had a lower chance of having no functional difficulties. It is possible that the disability declined dramatically between the two waves. Thus, when older women increased their protein intake, the intake was still not enough to compensate their physical functioning decline.

### 4.5. Limitations

There are several limitations to this study. First, some of the variables used to define successful aging and health-related behaviors were not available and were not consistent across the five waves of the IFLS. Only the latest two waves were suitable for application for this study. Furthermore, only consistent variables in the two waves were selected as measurements, such as dietary intake items and cognitive function. Second, only two waves were included for analysis, and the dynamic changes of behaviors between the 7 years were not detected. Third, the data were from self-reported surveys. The data of some variables might not have been accurate, such as morbidity from chronic diseases. However, self-reported survey is the most feasible method to obtain data from community-based people, and IFLS was a nationally representative source of longitudinal data in Indonesia. Such longitudinal data were very valuable in research of public health. Fourth, we defined successful aging indicators as binary variables (success or failure) due to the available measurement in this data. The definition may be too arbitrary and can not show a complete picture of successful aging. Fifth, the cutting point to define low, moderate, and high degree of physical activity measured by IPAQ was based on the criteria of general population but not for older people. The criteria may be too strict for older people.

## 5. Conclusions

Health-related behaviors and changes in those behaviors may affect successful aging in older people. However, changes in health behaviors may require a longer time and an early start to produce significant improvements in successful aging. We suggest promoting increasing physical activity, no smoking/smoking cessation, and assuring appropriate protein intake for Indonesian older adults to achieve successful aging. Friendly and accessible methods to provide health information about how to promote healthy behaviors for older people should be considered, such as through TV programs or health education activities held by the Posyandu Lansia or providing booklets about preparation of successful aging. Building up a healthy lifestyle should begin as early as possible, and then, the effects on successful aging would be more effective. Health promotion education and health literacy about healthy lifestyle should also be provided for younger generations in the school education for teenagers and beyond. In addition, gender differences exist in health behaviors and successful aging. We also suggest that a gender-sensitive intervention aimed at promoting healthy lifestyles for successful aging by Indonesian older adults should be conducted in the future.

## Figures and Tables

**Table 1:** The description of the sample of the IFLS wave 4 and wave 5 data for older people aged 60 and above (%).

Variables	Baseline (2007)	Follow-Up (2014)
Age		
Age 60–69	84.4	---
Age 70+	15.6	---
Sex		
Female	52.1	---
Male	47.9	---
Education		
College, university, and above	4.7	---
Senior high school	8.3	---
Junior high school	6.7	---
No education to elementary school	51.1	---
No formal education	29.2	---
Monthly expenditure at baseline		
USD 2.98~26.91	25.1	---
USD 26.92~39.62	22.0	---
USD 39.63~56.50	20.4	---
USD 56.51~91.45	18.7	---
USD 91.43+	13.7	---
Ethnicity		---
Javanese	48.6	---
Non-Javanese	51.4	---
Religion		
Islam	87.2	---
Others (Catholic, Protestant, Hindu, Buddhism)	12.9	---
Place of residence		
Urban	43.4	50.7
Rural	56.6	49.3
Health Insurance		
Yes	29.1	52.3
No	70.9	47.7
Health-related behaviors		
Smoking		
Yes	55.1	47.6
No	44.9	52.4
Physical activity		
Low	56.6	51.4
Medium	18.9	22.7
High	24.5	25.9
Protein intake		
High	61.8	53.7
low	38.2	46.3
Successful Aging		
Chronic disease numbers		
Having chronic disease	36.6	43.8
No chronic disease	63.4	56.2
Physical function		
Having physical difficulty	6.7	42.3
No physical function difficulty	93.3	57.7
Depressive symptoms		
Having depressive symptoms	2.1	16.5
No depressive symptoms	97.9	83.5
Cognitive function		
Impaired cognitive function	44.7	60.0
No cognitive impairment	55.3	40.0
Social support		
No social support	30.3	42.9
Having social support	69.7	57.1
Social participation		
No social participation	5.0	20.8
Having social participation	95.0	79.2
Overall Successful aging		
Failed	76.4	94.4
Successful	23.6	5.6

Note: *N* = 1289. Only the participants who completed both waves were included.

**Table 2:** Changes of health-related behaviors by sex.

Variables Baseline-Followup	Total (*n* = 1289)		Males (*n* = 617)		Females (*n* = 672)	
	*N*	%	*N*	%	*N*	%
Smoking	***					
No–No	616	47.8	82	13.3	534	79.5
No–Yes	99	7.7	58	9.4	41	6.1
Yes–No	67	5.2	38	6.2	29	4.3
Yes–Yes	507	39.3	439	71.2	68	10.1
Protein intake						
Low–Low	308	23.9	135	21.9	173	25.7
Low–High	184	14.3	99	16.0	85	12.6
High–Low	288	22.3	133	21.6	155	23.1
High–High	509	39.5	250	40.5	259	38.5
Physical activity	**					
Stable	517	40.5	223	36.6	294	44.1
Reduced	335	26.2	160	26.2	175	26.2
Increased	425	33.3	227	37.2	198	29.7

Note: Chi-square test was analyzed. ** *p* < 0.01, *** *p* < 0.001.

**Table 3:** Successful aging rate in the two waves by gender (%).

Successful Aging Indicators	Total	Men	Women
2007			
No chronic disease	63.4% ***	70.3%	57.1%
No physical function difficulty	93.3%	93.9%	92.8%
No depressive symptoms	97.9%	98.7%	97.2%
No cognitive impairment	55.3% ***	61.6%	49.1%
Having social support	69.7% ***	91.6%	49.7%
Having social participation	95.0% ***	90.3%	99.4%
Overall successful aging	23.6% ***	32.8%	14.7%
2014			
No chronic disease	56.2% ***	61.3%	51.5%
No physical function difficulty	57.7% ***	48.6%	66.1%
No depressive symptoms	83.5%	84.6%	82.4%
No cognitive impairment	40.0% ***	48.1%	32.6%
Having social support	57.1% ***	83.6%	32.7%
Having social participation	79.2% *	81.7%	76.9%
Overall successful aging	5.6% ***	8.9%	2.5%

Note: *N* = 1289. Analysis by Chi-square test, * *p* < 0.05, *** *p* < 0.001.

**Table 4:** Health-related behavior changes and factors related to longitudinal successful aging by logistic regression among male older adults (odds ratios and 95% confidence interval).

Variables at Baseline	No Chronic Disease	No Physical Difficulty	Intact Cognitive Function	No Depressive Symptoms	Having Social Support	Having Social Participation	Overall Successful Aging
Demographics							
Age							
Age 60–69	1	1	1	1	1	1	1
Age 70+	1.36 (0.82–2.27)	0.6 7(0.42–1.07)	0.25 (0.14–0.44) ***	1.10 (0.58–2.09)	0.66 (0.37–1.17)	0.55 (0.32–0.95) *	0.21 (0.04–0.89) *
Education at baseline	0.75 (0.61–0.91) **	0.93 (0.77–1.13)	1.97 (1.57–2.46) ***	1.22 (0.93–1.61)	0.97 (0.75–1.25)	1.44 (1.10–1.89) **	1.27 (0.92–1.75)
Monthly expenditure at baseline	0.85 (0.74–0.99) *	1.08 (0.94–1.24)	1.00 (0.86–1.16)	0.91 (0.75–1.10)	0.95 (0.79–1.15)	1.10 (0.92–1.32)	0.86 (0.67–1.11)
Place of residence at baseline							
Urban	1	1	1	1	1	1	1
Rural	0.98 (0.65–1.48)	0.89 (0.60–1.31)	0.81 (0.54–1.21)	0.65 (0.38–1.12)	1.06 (0.62–1.79)	1.38 (0.84–2.27)	1.04 (0.52–2.07)
Ethnicity							
Non-Javanese	1	1	1	1	1	1	1
Javanese	0.99 (0.68–1.43)	0.86 (0.61–1.22)	0.93 (0.64–1.35)	1.19 (0.74–1.92)	1.00 (0.62–1.60)	1.95 (1.22–3.10) **	1.09 (0.59–2.01)
Health insurance at baseline							
No	1	1	1	1	1	1	1
Yes	0.77 (0.47–1.28)	0.97 (0.60–1.56)	0.88 (0.52–1.47)	1.04 (0.52–2.05)	0.78 (0.43–1.41)	1.39 (0.73–2.68)	0.76 (0.32–1.76)
Demographic Changes							
Changes of residence							
Stable	1	1	1	1	1	1	1
Changed	1.05 (0.54–2.03)	1.69 (0.90–3.17)	1.11 (0.58–2.11)	0.95 (0.42–2.10)	0.46 (0.22–0.95) *	0.86 (0.39–1.89)	0.74 (0.21–2.61)
Changes of health insurance							
Stable	1	1	1	1	1	1	1
From no to yes	0.56 (0.36–0.86) **	0.98 (0.65–1.48)	1.25 (0.81–1.93)	0.78 (0.45–1.35)	2.26 (1.19–4.31) *	1.04 (0.61–1.76)	0.81 (0.38–1.73)
From yes to no	1.85 (0.83–4.11)	0.85 (0.42–1.72)	1.19 (0.56–2.52)	0.75 (0.29–1.93)	1.21 (0.51–2.87)	1.03 (0.39–2.69)	1.85 (0.58–5.86)
Health-Related Behavior and Changes							
Smoking at baseline							
No	1	1	1	1	1	1	1
Yes	0.67 (0.37–1.21)	1.13 (0.66–1.93)	1.13 (0.63–2.02)	1.24 (0.59–2.57)	0.21 (0.06–0.70) *	1.51 (0.76–3.01)	0.61 (0.27–1.36)
Smoking changes							
Stable and started smoking	1	1	1	1	1	1	1
Quitting smoking	0.41 (0.18–0.91) *	1.12 (0.54–2.35)	0.82 (0.37–1.82)	1.81 (0.60–5.41)	0.22 (0.55–0.89) *	1.49 (0.58–3.81)	0.36 (0.09–1.43)
Physical activity at baseline							
Low	1	1	1	1	1	1	1
Medium	1.83 (1.12–2.99) *	0.56 (0.36–0.88) *	1.38 (0.87–2.19)	1.47 (0.81–2.70)	1.29 (0.68–2.44)	1.50 (0.82–2.76)	1.51 (0.70–3.27)
High	1.67 (0.90–3.09)	0.58 (0.32–1.03)	1.81 (0.98–3.34)	1.33 (0.59–2.96)	0.65 (0.31–1.36)	1.25 (0.60–2.61)	0.98 (0.35–2.74)
Physical activity changes							
Stable	1	1	1	1	1	1	1
Reduced	0.73 (0.41–1.30)	1.52 (0.89–2.57)	0.75 (0.43–1.31)	1.23 (0.58–2.61)	1.02 (0.51–2.04)	0.83 (0.42–1.63)	0.87 (0.35–2.15)
Increased	1.33(0.87–2.05)	0.93(0.62–1.39)	0.97(0.63–1.49)	1.04(0.61–1.78)	1.17(0.66–2.07)	1.98(1.15–3.42) *	0.71(0.34–1.44)
Protein intake changes							
Low stable	1	1	1	1	1	1	1
Low to high	0.84 (0.46–1.55)	2.18 (1.24–3.83) **	1.17 (0.64–2.12)	1.32 (0.60–2.89)	0.93 (0.44–1.94)	0.91 (0.44–1.87)	1.19 (0.41–3.45)
High to low	1.09 (0.62–1.92)	1.12 (0.67–1.86)	1.09 (0.63–1.88)	0.86 (0.44–1.68)	0.76 (0.39–1.49)	0.99 (0.50–1.95)	1.41 (0.55–3.60)
High stable	0.65 (0.39–1.09)	1.14 (0.71–1.84)	1.28 (0.77–2.13)	1.23 (0.64–2.35)	1.32 (0.68–2.56)	0.87 (0.46–1.61)	1.65 (0.68–4.00)

Note: Binary logistic regression was used for analysis. The reference group of the variables: chronic disease (have chronic disease), physical function (have physical function), depressive symptoms (have depressive symptoms), cognitive (have impaired cognitive function), social support (not having), social participation (not having), overall successful aging (failed), age (age 60–69), gender (women), monthly expenditure, residence (urban), ethnicity (Javanese), health insurance (yes), smoking (no), protein intake and changes (low stable), physical activity (low), changes of health insurance (stable), changes of residence (stable), physical activity changes (stable), and smoking changes (stable and starting smoking). * *p* < 0.05, ** *p* < 0.01, *** *p* < 0.001.

**Table 5:** Health-related behavior changes and factors related to longitudinal successful aging by logistic regression among female older adults (odds ratios and 95% confidence interval).

Variables	No Chronic Disease	No Physical Difficulty	Intact Cognitive Function	No Depressive Symptoms	Having Social Support	Having Social Participation	Overall Successful Aging
Demographics							
Age at baseline							
Age 60–69	1	1	1	1	1	1	1
Age 70+	1.02 (0.64–1.60)	0.69 (0.44–1.09)	0.33 (0.17–0.67) **	1.16 (0.63–2.10)	0.35 (0.20–0.64) **	1.13 (0.67–1.91)	<0.01 (0.00–0.00)
Education at baseline	0.84 (0.68–1.04)	1.05 (0.85–1.30)	2.52 (1.94–3.27) ***	1.08 (0.83–1.41)	1.07 (0.86–1.33)	1.72 (1.27–2.34) ***	2.24 (1.25–4.01) **
Monthly expenditure at baseline	0.85 (0.75–0.96) *	1.03 (0.90–1.18)	1.14 (0.98–1.32)	1.00 (0.84–1.17)	0.91(0.79–1.04)	1.11 (0.95–1.30)	0.79 (0.50–1.25)
Place of residence at baseline							
Urban	1	1	1	1	1	1	1
Rural	1.18 (0.83–1.69)	1.06 (0.73–1.54)	0.78 (0.52–1.18)	0.89 (0.56–1.43)	1.29 (0.88–1.90)	0.74 (0.48–1.14)	0.89 (0.23–3.34)
Ethnicity							
Non-Javanese	1	1	1	1	1	1	1
Javanese	1.39 (0.99–1.95)	0.95 (0.67–1.34)	0.78 (0.52–1.17)	1.28 (0.82–1.98)	1.70 (1.19–2.45) **	2.23 (1.47–3.39) ***	0.52 (0.16–1.71)
Health insurance at baseline							
No	1	1	1	1	1	1	1
Yes	0.84 (0.53–1.32)	1.26 (0.78–2.02)	0.62 (0.95–2.74)	0.93 (0.53–1.65)	1.10 (0.67–1.79)	1.46 (0.80–2.67)	0.93 (0.20–4.18)
Demographic Changes							
Changes of residence							
Stable	1	1	1	1	1	1	1
Changed	0.14 (0.82–2.60)	0.96 (0.53–1.75)	0.73 (0.36–1.46)	0.66 (0.34–1.28)	1.29 (0.71–2.33)	1.95 (0.93–4.06)	4.51 (1.05–19.25) *
Changes of health insurance							
Stable	1	1	1	1	1	1	1
From no to yes	0.94 (0.63–1.40)	1.37 (0.90–2.08)	1.55 (0.98–2.43)	1.42 (0.84–2.42)	1.32 (0.87–2.01)	0.97 (0.61–1.55)	0.76 (0.18–3.16)
From yes to no	2.02 (1.01–4.02) *	1.08 (0.53–2.19)	0.66 (0.30–1.45)	1.93 (0.73–5.12)	0.66 (0.31–1.42)	0.75 (0.31–1.78)	1.78 (0.26–12.01)
Health-Related Behavior and changes							
Smoking at baseline							
No	1	1	1	1	1	1	1
Yes	0.89 (0.55–1.43)	0.95 (0.58–1.53)	0.99 (0.55–1.76)	0.9 4(0.51–1.72)	0.70 (0.41–1.18)	0.69 (0.41–1.17)	0.68 (0.07–6.01)
Smoking changes							
Stable and started smoking	1	1	1	1	1	1	1
Quitting smoking	0.99 (0.50–1.93)	1.84 (0.86–3.93)	0.95 (0.41–2.22)	1.07 (0.45–2.53)	0.44 (0.18–1.04)	1.19 (0.54–2.62)	3.85 (0.84–17.48)
Physical activity at baseline							
Low	1	1	1	1	1	1	1
Medium	1.87 (0.94–3.70)	1.04 (0.52–2.06)	0.98 (0.44–2.19)	0.86 (0.37–2.00)	1.01 (0.48–2.10)	1.59 (0.71–3.58)	1.36 (0.08–21.42)
High	1.45 (0.76–2.75)	1.03 (0.54–1.99)	1.22 (0.58–2.59)	0.78 (0.35–1.71)	1.58 (0.82–3.06)	1.27 (0.59–2.75)	2.79 (0.24–32.31)
Physical activity changes							
Stable	1	1	1	1	1	1	1
Reduced	0.97 (0.51–1.84)	0.98 (0.51–1.87)	1.36 (0.64–2.89)	1.31 (0.60–2.86)	0.62 (0.32–1.20)	1.32 (0.61–2.87)	2.81 (0.30–26.21)
Increased	1.45 (0.98–2.13)	1.21 (0.81–1.81)	1.75 (1.11–2.76) *	1.28 (0.77–2.13)	1.20 (0.80–1.82)	1.79 (1.12–2.84) *	2.80 (0.53–14.67)
Protein intake and changes							
Low stable	1	1	1	1	1	1	1
Low to high	0.80 (0.45–1.42)	0.50 (0.28–0.89) *	0.66 (0.32–1.35)	1.05 (0.49–2.62)	1.19 (0.64–2.20)	1.50 (0.75–3.00)	0.66 (0.09–4.83)
High to low	1.29 (0.81–2.05)	0.82 (0.50–1.33)	1.09 (0.62–1.89)	1.10 (0.59–2.05)	1.15 (0.70–1.90)	1.28 (0.75–2.19)	0.67 (0.10–4.20)
High stable	0.84 (0.54–1.30)	0.71 (0.45–1.13)	1.04 (0.62–1.76)	0.75 (0.43–1.32)	1.21 (0.75–1.93)	1.44 (0.86–2.42)	0.80 (0.17–3.65)

Note: Binary logistic regression was used for analysis. The reference group of the variables: chronic disease (have chronic disease), physical function (have physical function), depressive symptoms (have depressive symptoms), cognitive (have impaired cognitive function), social support (not having), social participation (not having), overall successful aging (failed), age (age 60–69), gender (women), education, monthly expenditure, residence (urban), ethnicity (Javanese), health insurance (yes), smoking (no), protein intake and changes (low stable), physical activity (low), changes of health insurance (stable), changes of residence (stable), physical activity changes (stable), and smoking changes (stable and starting smoking). * *p* < 0.05, ** *p* < 0.01, *** *p* < 0.001.

## Supplementary Materials

The following supporting information can be downloaded at: https://www.mdpi.com/article/10.3390/ijerph19105952/s1, Table S1: Bivariate analysis for successful aging by related factors among male older adults in 2014; Table S2: Bivariate analysis for successful aging by related factors among female older adults in 2014.
