# Supplementary material for: Effects of Health-Related Behaviors and Changes on Successful Aging among Indonesian Older People
Source: Int J Environ Res Public Health. 2022 May 13;19(10):5952. doi: 10.3390/ijerph19105952 (PMC9141271; doi:10.3390/ijerph19105952)
Supplement: Supplementary file 1 [file ijerph-19-05952-s001.zip › ijerph-1709820-supplementary.pdf]

Supplementary Materials

Table S1. Bivariate analysis for successful aging by related factors among male older adults in 2014

Variables at Baseline and the changes between two waves	Successful aging in the follow-up																				
	Chronic disease		*P* Value	Physical function difficulty		*P* Value	Cognitive function		*P* Value	Depression		*P* Value	Social support		*P* Value	Social participation		*P* Value	Overall Successful Aging		*P* Value
	Any	None		Having difficulty	No difficulty		Impaired	Not impaired		Depressed	Not depressed		No	Yes		No	Yes		Failed	Successful	
Total	38.7%	61.3%		51.4%	48.6%		41.9%	48.1%		15.4%	84.6%		16.4%	83.6%		18.3%	81.7%		91.1%	8.9%	
Demographics																					
Age at baseline			0.249			0.210			0.000			1.000			0.048			0.008			0.017
Age 60-69	39.8%	60.2%		50.2%	49.8%		46.7%	53.3%		15.4%	84.6%		15.0%	85.0%		16.5%	83.5%		89.8%	10.2%	
Age 70+	33.0%	67.0%		57.7%	42.3%		79.4%	20.6%		15.5%	84.5%		23.7%	76.3%		27.8%	72.2%		97.9%	2.1%	
Education at baseline			0.000			0.315			0.000			0.061			0.378			0.005			0.070
No formal education	25.8%	74.2%		60.2%	39.8%		77.4%	22.6%		14.0%	86.0%		20.4%	79.6%		30.1%	69.9%		98.9%	1.1%	
elementary school	35.1%	64.9%		49.0%	51.0%		56.7%	43.3%		18.1%	81.9%		14.5%	85.5%		18.1%	81.9%		90.1%	9.9%	
Junior high school	46.7%	53.3%		53.3%	46.7%		26.7%	73.3%		13.3%	86.7%		22.2%	77.8%		17.8%	82.2%		88.9%	11.1%	
Senior high school	53.0%	47.0%		56.3%	43.8%		31.8%	68.2%		13.6%	86.4%		19.7%	80.3%		12.1%	87.9%		87.9%	12.1%	
College, university, and above	64.6%	35.4%					16.7%	83.3%		2.1%	97.9%		12.5%	87.5%		6.3%	93.8%		89.6%	10.4%	
Monthly expenditur			0.000			0.281			0.013			0.796			0.963			0.296			0.933

e at baseline																					
$2.98~26.91	28.0%	72.0%		56.1%	43.9%		61.0%	39.0%		14.0%	86.0%		15.9%	84.1%		23.2%	76.8%		90.2%	9.8%	
$26.92~39.62	28.2%	71.8%		54.2%	45.8%		57.0%	43.0%		17.6%	82.4%		16.9%	83.1%		16.9%	83.1%		92.3%	7.7%	
$39.63~56.50	44.2%	55.8%		43.4%	56.6%		49.6%	50.4%		14.2%	85.8%		17.7%	82.3%		17.7%	82.3%		90.3%	9.7%	
$56.51~91.45	50.0%	50.0%		50.9%	49.1%		45.4%	54.6%		18.5%	81.5%		14.8%	85.2%		18.5%	81.5%		92.6%	7.4%	
$91.43+	53.9%	46.1%		48.7%	51.3%		40.8%	59.2%		14.5%	85.5%		18.4%	81.6%		11.8%	88.2%		92.1%	7.9%	
Place of Residence at baseline			0.002			0.367			0.000			0.079			0.474			0.966			0.825
Urban	46.3%	53.7%		49.0%	51.0%		42.0%	58.0%		12.2%	87.8%		14.9%	85.1%		18.0%	82.0%		90.6%	9.4%	
Rural	33.4%	66.6%		53.0%	47.0%		58.8%	41.2%		17.7%	82.3%		17.4%	82.6%		18.5%	81.5%		91.4%	8.6%	
Changes of residence			0.982			0.245			0.731			0.462			0.016			0.090			0.845
Stable	38.8%	61.2%		52.4%	47.6%		51.7%	48.3%		15.0%	85.0%		15.4%	84.6%		18.2%	81.8%		91.0%	9.0%	
Rural to urban	38.8%	61.2%		40.8%	59.2%		55.1%	44.9%		20.4%	79.6%		24.5%	75.5%		16.3%	83.7%		91.8%	8.2%	
Urban to rural	33.3%	66.7%		33.3%	66.7%		33.3%	66.7%		0.0%	100.0%		66.7%	33.3%		66.7%	33.3%		100.0%	0.0%	
Health insurance at baseline			0.307			0.959			0.101			0.478			0.049			0.092			0.829
No	36.6%	63.4%		51.3%	48.7%		54.4%	45.6%		16.1%	83.9%		14.2%	85.8%		20.1%	79.9%		91.3%	8.7%	
Yes	41.4%	58.6%		51.1%	48.9%		46.8%	53.2%		13.4%	86.6%		21.0%	79.0%		14.0%	86.0%		90.3%	9.7%	
Changes of health insurance			0.530			0.822			0.530			0.514			0.014			0.919			0.713
Stable	52.7%	47.3%		51.1%	48.9%		52.7%	47.3%		13.9%	86.1%		18.7%	81.3%		18.2%	81.8%		91.1%	8.9%	

From no to yes	48.8%	51.2%		50.0%	50.0%		48.8%	51.2%		17.1%	82.9%		9.1%	90.9%		18.9%	81.1%		91.5%	8.5%	
From yes to no	57.1%	42.9%		55.1%	44.9%		57.1%	42.9%		18.4%	81.6%		20.4%	79.6%		16.3%	83.7%		87.8%	12.2%	
Ethnicity			0.341			0.087			0.504			0.419			1.000			0.004			0.897
Non-Javanese	40.2%	59.8%		47.6%	52.4%		50.5%	49.5%		16.4%	83.6%		16.1%	83.9%		22.8%	77.2%		90.7%	9.3%	
Javanese	36.1%	63.9%		54.8%	45.2%		53.5%	46.5%		14.0%	86.0%		16.4%	83.6%		13.4%	86.6%		91.3%	8.7%	
Health Behavior																					
Protein Intake changes			0.001			0.101			0.033			0.731			0.306			0.946			0.564
High stable	47.6%	52.4%		51.2%	48.8%		46.0%	54.0%		14.0%	86.0%		13.2%	86.8%		18.8%	81.2%		90.0%	10.0%	
High to low	32.3%	67.7%		52.6%	47.4%		51.1%	48.9%		18.0%	82.0%		20.3%	79.7%		16.5%	83.5%		89.5%	10.5%	
Low to high	38.4%	61.6%		41.4%	58.6%		54.5%	45.5%		14.1%	85.9%		18.2%	81.8%		19.2%	80.8%		92.9%	7.1%	
Low stable	28.9%	71.1%		57.8%	42.2%		61.5%	38.5%		16.3%	83.7%		17.0%	83.0%		18.5%	81.5%		93.3%	6.7%	
Physical Activity at baseline			0.001			0.100			0.234			0.283			0.045			0.439			0.690
Low	44.3%	55.7%		47.3%	52.7%		51.7%	48.3%		17.6%	82.4%		15.5%	84.5%		18.9%	81.1%		91.2%	8.8%	
Medium	27.3%	72.7%		57.4%	42.6%		56.3%	43.7%		13.7%	86.3%		12.6%	87.4%		15.3%	84.7%		89.6%	10.4%	
High	39.7%	60.3%		51.1%	48.9%		46.6%	53.4%		12.2%	87.8%		22.9%	77.1%		20.6%	79.4%		92.4%	7.6%	
Physical activity changes			0.273			0.705			0.983			0.384			0.124			0.014			0.850
Stable	41.7%	58.3%		50.2%	49.8%		51.6%	48.4%		16.6%	83.4%		17.9%	82.1%		22.4%	77.6%		90.1%	9.9%	
Reduced	38.8%	61.3%		49.4%	50.6%		51.9%	48.1%		11.9%	88.1%		19.4%	80.6%		20.6%	79.4%		91.3%	8.8%	
Increased	34.4%	65.6%		53.3%	46.7%		52.4%	47.6%		16.3%	83.7%		12.3%	87.7%		12.3%	87.7%		91.6%	8.4%	

Smoking at baseline			0.079			0.926			0.163			0.371			0.011			0.940			0.606
No	45.1%	54.9%		51.9%	48.1%		46.6%	53.4%		12.8%	87.2%		9.0%	91.0%		18.8%	81.2%		89.5%	10.5%	
Yes	36.3%	63.7%		50.9%	49.1%		53.5%	46.5%		15.9%	84.1%		18.2%	81.8%		18.0%	82.0%		91.4%	8.6%	
Smoking changes			0.017			0.889			0.869			0.551			0.342			0.198			0.365
Stable	37.4%	62.6%		51.4%	48.6%		51.6%	48.4%		15.8%	84.2%		16.9%	83.1%		17.3%	82.7%		90.3%	9.7%	
Started smoking	26.3%	73.7%		52.6%	47.4%		52.6%	47.4%		15.8%	84.2%		7.9%	92.1%		28.9%	71.1%		94.7%	5.3%	
Quitted smoking	53.4%	46.6%		48.3%	51.7%		55.2%	44.8%		10.3%	89.7%		15.5%	84.5%		19.0%	81.0%		94.8%	5.2%	

Note: N=617. Analysis by Chi-square test.

Table S2. Bivariate analysis for successful aging by related factors among female older adults in 2014

Variables at Baseline and the changes between two waves	Successful aging in the follow-up																				
	Chronic disease		*P* Value	Physical function		*P* Value	Cognitive function		*P* Value	Depression		*P* Value	Social support		*P* Value	Social participation		*P* Value	Overall Successful Aging		*P* Value
	Any	None		Having difficulty	No difficulty		Impaired	Not impaired		Depressed	Not depressed		No	Yes		No	Yes		Failed	Successful	
Total	48.5%	51.5%		33.9%	66.1%		67.4%	32.6%		17.6%	82.4%		67.3%	32.7%		23.1%	76.9%		97.5%	2.5%	
Demographics																					
Age at baseline			0.399			0.064			0.000			0.831			0.000			0.525			0.074
Age 60-69	49.3%	50.7%		32.4%	67.6%		63.6%	36.4%		17.8%	82.2%		64.3%	35.7%		22.5%	77.5%		97.0%	3.0%	
Age 70+	44.2%	55.8%		42.3%	57.7%		88.5%	11.5%		16.3%	83.7%		83.7%	16.3%		26.0%	74.0%		100.0%	0.0%	
Education at baseline			0.009			0.703			0.000			0.221			0.153			0.000			0.000
No formal education	41.9%	58.1%		36.3%	63.7%		84.5%	15.5%		17.3%	82.7%		70.8%	29.2%		32.7%	67.3%		99.3%	0.7%	
elementary school	50.7%	49.3%		33.3%	66.7%		62.9%	37.1%		19.4%	80.6%		65.3%	34.7%		18.4%	81.6%		97.3%	2.7%	
Junior high school	56.1%	43.9%		26.8%	73.2%		48.8%	51.2%		17.1%	82.9%		73.2%	26.8%		12.2%	87.8%		87.8%	12.2%	
Senior high school	63.4%	36.6%		31.7%	68.3%		17.1%	82.9%		4.9%	95.1%		56.1%	43.9%		2.4%	97.6%		100.0%	0.0%	
College, university, and above	75.0%	25.0%		25.0%	75.0%		8.3%	91.7%		25.0%	75.0%		50.0%	50.0%		16.7%	83.3%				
Monthly expenditure at baseline			0.003			0.081			0.000			0.960			0.737			0.001			0.529

$2.98~26.91	37.3%	62.7%		39.2%	60.8%		81.0%	19.0%		16.3%	83.7%		65.4%	34.6%		35.9%	64.1%		97.4%	2.6%	
$26.92~39.62	45.6%	54.4%		25.0%	75.0%		71.3%	28.7%		17.6%	82.4%		66.9%	33.1%		21.3%	78.7%		97.1%	2.9%	
$39.63~56.50	50.0%	50.0%		38.2%	61.8%		72.9%	27.1%		19.4%	80.6%		67.4%	32.6%		20.1%	79.9%		99.3%	0.7%	
$56.51~91.45	52.3%	47.7%		34.4%	65.6%		57.8%	42.2%		16.4%	83.6%		72.7%	27.3%		16.4%	83.6%		97.7%	2.3%	
$91.43+	61.9%	38.1%		30.9%	69.1%		47.4%	52.6%		17.5%	82.5%		66.0%	34.0%		20.6%	79.4%		95.9%	4.1%	
Place of Residence at baseline			0.003			1.000			0.000			0.320			0.506			0.001			0.518
Urban	54.9%	45.1%		33.9%	66.1%		57.2%	42.8%		15.8%	84.2%		68.8%	31.3%		17.1%	82.9%		97.0%	3.0%	
Rural	43.2%	56.8%		34.0%	66.0%		75.8%	24.2%		19.0%	81.0%		66.0%	34.0%		28.0%	72.0%		97.8%	2.2%	
Changes of residence			0.560			0.980			0.588			0.142			0.667			0.337			0.028
Stable	49.1%	50.9%		34.0%	66.0%		66.9%	33.1%		16.7%	83.3%		67.8%	32.2%		23.6%	76.4%		97.9%	2.1%	
Rural to urban	43.6%	56.4%		32.7%	67.3%		70.9%	29.1%		27.3%	72.7%		61.8%	38.2%		20.0%	80.0%		94.5%	5.5%	
Urban to rural	33.3%	66.7%		33.3%	66.7%		83.3%	16.7%		16.7%	83.3%		66.7%	33.3%		0.0%	100.0%		83.3%	16.7%	
Health insurance at baseline			0.492			0.445			0.002			0.741			0.686			0.011			0.327
No	47.3%	52.7%		34.9%	65.1%		71.4%	28.6%		17.8%	82.2%		67.0%	33.0%		25.9%	74.1%		97.9%	2.1%	
Yes	50.3%	49.7%		31.4%	68.6%		58.4%	41.6%		16.8%	83.2%		68.6%	31.4%		16.2%	83.8%		96.2%	3.8%	
Changes of health insurance			0.275			0.352			0.275			0.111			0.176			0.993			0.836
Stable	69.4%	30.6%		35.8%	64.2%		69.4%	30.6%		19.7%	80.3%		68.5%	31.5%		23.4%	76.6%		97.5%	2.5%	
From no to yes	62.8%	37.2%		30.2%	69.8%		62.8%	37.2%		14.0%	86.0%		62.8%	37.2%		23.3%	76.7%		97.7%	2.3%	

From yes to no	69.8%	30.2%		30.2%	69.8%		69.8%	30.2%		11.3%	88.7%		75.5%	24.5%		22.6%	77.4%		96.2%	3.8%	
Ethnicity			0.023			0.943			0.444			0.192			0.007			0.000			0.281
Non-Javanese	52.6%	47.4%		34.1%	65.9%		66.2%	33.8%		19.4%	80.6%		72.3%	27.7%		28.9%	71.1%		96.8%	3.2%	
Javanese	43.5%	56.5%		33.5%	66.5%		69.3%	30.7%		15.5%	84.5%		62.1%	37.9%		17.1%	82.9%		98.1%	1.9%	
Health Behavior																					
Protein Intake changes			0.005			0.236			0.007			0.458			1.000			0.169			0.583
High stable	54.8%	45.2%		34.4%	65.6%		59.8%	40.2%		20.5%	79.5%		67.2%	32.8%		20.1%	79.9%		96.5%	3.5%	
High to low	40.6%	59.4%		33.5%	66.5%		70.3%	29.7%		14.8%	85.2%		67.1%	32.9%		24.5%	75.5%		98.7%	1.3%	
Low to high	56.5%	43.5%		42.4%	57.6%		69.4%	30.6%		16.5%	83.5%		67.1%	32.9%		18.8%	81.2%		97.6%	2.4%	
Low stable	42.2%	57.8%		29.5%	70.5%		75.1%	24.9%		16.2%	83.8%		67.6%	32.4%		28.3%	71.7%		97.7%	2.3%	
Physical Activity at baseline			0.099			0.960			0.339			0.893			0.521			0.239			0.055
Low	50.6%	49.4%		33.5%	66.5%		68.4%	31.6%		17.1%	82.9%		67.0%	33.0%		25.3%	74.7%		98.4%	1.6%	
Medium	36.2%	63.8%		34.5%	65.5%		74.1%	25.9%		17.2%	82.8%		74.1%	25.9%		20.7%	79.3%		98.3%	1.7%	
High	46.2%	53.8%		34.6%	65.4%		64.3%	35.7%		18.7%	81.3%		66.5%	33.5%		19.2%	80.8%		95.1%	4.9%	
Physical activity changes			0.076			0.660			0.189			0.536			0.507			0.014			0.023
Stable	53.1%	46.9%		35.0%	65.0%		71.4%	28.6%		19.0%	81.0%		67.7%	32.3%		28.6%	71.4%		99.0%	1.0%	
Reduced	44.6%	55.4%		34.9%	65.1%		65.7%	34.3%		17.7%	82.3%		70.3%	29.7%		18.3%	81.7%		94.9%	5.1%	
Increased	43.9%	56.1%		31.3%	68.7%		64.1%	35.9%		15.2%	84.8%		64.6%	35.4%		19.7%	80.3%		97.5%	2.5%	
Smoking at baseline			0.358			0.541			0.039			0.776			0.236			0.121			0.498

No	48.9%	51.1%		33.3%	66.7%		66.1%	33.9%		17.4%	82.6%		66.5%	33.5%		22.1%	77.9%		97.2%	2.8%	
Yes	43.3%	56.7%		37.1%	62.9%		77.3%	22.7%		18.6%	81.4%		73.2%	26.8%		29.9%	70.1%		99.0%	1.0%	
Smoking changes			0.509			0.300			0.101			0.576			0.089			0.833			1.000
Stable	48.7%	51.3%		34.8%	65.2%		66.5%	33.5%		17.9%	82.1%		66.3%	33.7%		22.9%	77.1%		97.7%	2.3%	
Started smoking	37.9%	62.1%		27.6%	72.4%		82.8%	17.2%		10.3%	89.7%		69.0%	31.0%		27.6%	72.4%		100.0%	0.0%	
Quitted smoking	46.3%	53.7%		24.4%	75.6%		75.6%	24.4%		17.1%	82.9%		82.9%	17.1%		24.4%	75.6%		92.7%	7.3%	

Note: N=672. Analysis by Chi-square test.

Table S3. Moderating effects of age and education for health-related behavior changes and factors related to longitudinal successful aging by logistic regression among male older adults (odds ratios and 95% confidence interval)

Variables At Baseline	No chronic disease	No Physical difficulty	Intact cognitive function	No Depressive symptoms	Having social support	Having social participation	Overall Successful Aging
Demographics							
Age							
Age 60-69	1	1	1	1	1	1	1
Age 70+	1.43(0.35-5.83)	0.90(0.24-3.27)	0.03(0.00-0.40)**	1.63(1.75-15.23)	4.78(0.40-56.26)	0.30(0.06-1.350	<0.01(0.00-0.00)
Education at baseline	0.79(0.551.14)	0.79(0.56-1.12)	2.33(1.46-3.72)***	1.09(0.66-1.79)	0.88(0.49-1.56)	1.49(0.91-2.44)	1.46(0.81-2.61)
Monthly expenditure at baseline	0.85(0.73-0.98)*	1.08(0.93-1.24)	0.998(0.86-1.15)	0.91(0.75-1.10)	0.95(0.78-1.15)	1.11(0.92-1.34)	0.87(0.67-1.12)
Place of Residence at baseline							
Urban	1	1	1	1	1	1	1
Rural	0.97(0.64-1.46)	0.89(0.60-1.32)	0.83(0.55-1.26)	0.64(0.37-1.11)	1.08(0.63-1.84)	1.36(0.82-2.23)	1.02(0.51-2.06)
Ethnicity							
Non-Javanese	1	1	1	1	1	1	1
Javanese	0.98(0.67-1.42)	0.85(0.60-1.21)	0.87(0.59-1.27)	1.22(0.75-1.98)	0.97(0.60-1.56)	1.96(1.22-3.13)**	1.07(0.58-1.99)
Health insurance at baseline							
No	1	1	1	1	1	1	1
Yes	0.75(0.45-1.26)	1.00(0.62-1.63)	0.83(0.49-1.41)	1.03(0.51-2.08)	0.79(0.43-1.45)	1.35(0.70-2.63)	0.79(0.33-1.88)
Demographic changes							
Changes of residence							
Stable	1	1	1	1	1	1	1
Changed	1.07(0.55-2.09)	1.66(0.87-3.15)	1.22(0.63-2.34)	0.93(0.41-2.08)	0.47(0.23-0.99)*	0.81(0.36-1.79)	0.64(0.17-2.37)
Changes of health insurance							
Stable	1	1	1	1	1	1	1
From no to yes	0.54(0.35-0.85)**	0.97(0.64-1.48)	1.32(0.84-2.06)	0.77(0.44-1.36)	2.45(1.26-4.74)**	1.00(0.58-1.71)	0.75(0.35-1.63)
From yes to no	1.93(0.86-4.32)	0.82(0.40-1.68)	1.25(0.58-2.69)	0.73(0.28-1.90)	1.26(0.52-3.05)	1.02(0.39-2.71)	1.92(0.59-6.21)
Health-Related Behavior and Changes							
Smoking at baseline							
No	1	1	1	1	1	1	1
Yes	0.93(0.36-2.38)	1.01(0.43-2.21)	1.11(0.42-2.97)	1.13(0.35-3.59)	0.16(0.03-0.91)*	1.85(0.67-5.08)	1.25(0.29-5.25)

Smoking changes							
Stable & started smoking	1	1	1	1	1	1	1
Quitting smoking	0.40(0.17-0.93)*	0.49(0.23-1.04)	1.02(0.43-2.43)	1.63(0.53-4.99)	0.17(0.04-0.73)*	1.56(0.59-4.14)	0.39(0.09-1.64)
Physical Activity at baseline							
Low	1	1	1	1	1	1	1
Medium	1.68(0.73-3.89)	0.49(0.23-1.04)	3.40(1.52-7.55)**	1.20(0.42-3.38)	1.54(0.53-4.49)	0.88(0.32-2.40)	0.85(0.23-3.13)
High	1.27(0.51-3.14)	0.62(0.26-1.45)	4.09(1.64-10.18)**	0.64(0.19-2.12)	1.09(0.35-3.33)	1.40(0.49-4.00)	1.63(0.36-7.35)
Physical activity changes							
Stable	1	1	1	1	1	1	1
Reduced	0.71(0.40-1.27)	1.54(0.90-2.65)	0.76(0.43-1.34)	1.13(0.52-2.42)	1.01(0.49-2.07)	0.83(0.41-1.65)	0.86(0.34-2.15)
Increased	1.28(0.83-1.99)	0.95(0.63-1.43)	1.07(0.69-1.68)	0.95(0.55-1.64)	1.21(0.68-2.16)	1.96(1.12-3.43)*	0.72(0.35-1.50)
Protein intake changes							
Low stable	1	1	1	1	1	1	1
Low to high	0.82(0.44-1.52)	2.10(1.19-3.72)*	1.20(0.63-2.26)	1.35(0.61-2.96)	0.90(0.42-1.91)	0.91(0.44-1.88)	0.99(0.33-2.97)
High to low	1.23(0.58-2.62)	1.77(0.89-3.52)	2.29(1.02-5.14)*	0.74(0.29-1.83)	0.83(0.33-2.06)	1.41(0.57-3.44)	2.10(0.61-7.21)
High stable	0.74(0.36-1.55)	1.89(0.95-3.74)	2.64(1.19-5.84)*	1.04(0.42-2.60)	1.49(0.58-3.82)	1.28(0.54-3.05)	2.60(0.76-8.85)
Age (70+)*Smoking(no)	1	1	1	1	1	1	1
Age (70+)*Smoking(yes)	0.52(0.12-2.17)	0.57(1.61-2.03)	11.48(1.01-129.82)*	0.39(0.04-3.81)	0.19(0.01-2.06)	0.87(0.19-3.96)	20723558.17(0.00-0.00)
Age(70+)*PA(low)	1	1	1	1	1	1	1
Age(70+)*PA(medium)	2.53(0.56-11.30)	1.22(0.39-3.81)	1.17(0.0.28-4.85)	2.96(0.51-17.05)	1.16(0.23-5.92)	3.47(0.73-16.41)	1.34(0.66-27.15)
Age(70+)*PA(high)	1.01(0.30-3.42)	0.82(0.25-2.68)	0.86(0.20-3.57)	3.29(0.52-20.56)	0.25(0.06-1.02)	1.72(0.46-6.32)	0.00(0.00-.)
Age(70+)*Protein (low)	1	1	1	1	1	1	1
Age(70+)*Protein (high)	2.13(0.70-6.84)	1.55(0.59-4.09)	0.44(0.11-1.63)	0.96(0.25-3.62)	0.89(0.26-3.00)	2.39(0.75-7.65)	<0.01(0.00-0.00)
Education*Smoking(no)	1	1	1	1	1	1	1
Education*Smoking(yes)	0.85(0.58-1.26)	1.08(0.75-1.56)	0.98(0.60-1.60)	1.09(0.63-1.89)	1.17(0.66-2.07)	0.88(0.52-1.49)	0.64(0.34-1.21)
Education*PA(low)	1	1	1	1	1	1	1
Education*PA(medium)	0.99(0.55-1.78)	1.11(0.65-1.89)	0.44(0.24-0.79)**	1.06(0.47-2.37)	0.85(0.41-1.77)	1.51(0.64-3.59)	1.68(0.75-3.75)
Education*PA(high)	1.20(0.80-1.80)	0.97(0.65-1.43)	0.59(0.36-0.95)*	1.58(0.75-3.31)	0.87(0.53-1.42)	0.83(0.49-1.41)	0.75(0.35-1.62)
Education*Protein(low)	1	1	1	1	1	1	1
Education*Protein(high)	1.02(0.69-1.52)	1.44(0.98-2.11)	2.06(1.18-3.59)*	0.85(0.49-1.47)	1.10(0.67-1.82)	1.26(0.70-2.25)	1.56(0.83- 2.94)

Note: Binary logistic regression was used for analysis. The reference group of the variables: chronic disease (have chronic disease), physical function (have physical function), depressive symptoms (have depressive symptoms), cognitive (have impaired cognitive function), social support (no having), social participation (no having), overall successful aging (failed), age (age 60-69), gender (women), monthly expenditure, residence (urban), ethnicity (Javanese), health insurance (yes), smoking (no), protein intake &

changes (low stable), physical activity (low), changes of health insurance (stable), changes of residence (stable), physical activity changes (stable), smoking changes (stable & starting smoking). *p<0.05, **p<0.01, ***p<0.001.

Table S4. Moderating effects of age and education for health-related behavior changes and factors related to longitudinal successful aging by logistic regression among female older adults (odds ratios and 95% confidence interval)

Variables At Baseline	No chronic disease	No Physical difficulty	Intact cognitive function	No Depressive symptoms	Having social support	Having social participation	Overall Successful Aging
Demographics							
Age							
Age 60-69	1	1	1	1	1	1	1
Age 70+	0.67(0.34-1.35)	0.75(0.38-1.49)	0.31(0.11-0.91)*	0.93 (0.40-2.19)	0.28 (0.11-0.68)**	1.39 (0.62-3.09)	<0.01 (0.00-0.00)
Education at baseline	0.80(0.60-1.06)	0.84 (0.64-1.12)	2.46(1.72-3.52)***	1.22 (0.84-1.76)	0.90 (0.66-1.21)	1.52 (1.01-2.28)*	1.45 (0.56-3.73)
Monthly expenditure at baseline	0.84(0.74-0.96)*	1.05 (0.92-1.20)	1.14 (0.98-1.33)	0.99 (0.84-1.17)	0.92 (0.80-1.06)	1.13 (0.97-1.33)	0.74 (0.46-1.19)
Place of Residence at baseline							
Urban	1	1	1	1	1	1	1
Rural	1.19(0.83-1.71)	1.08 (0.74-1.57)	0.80 (0.52-1.21)	0.89 (0.55-1.42)	1.31 (0.89-1.93)	0.75 (0.48-1.15)	0.76 (0.19-2.93)
Ethnicity							
Non-Javanese	1	1	1	1	1	1	1
Javanese	1.42(1.01-2.00)*	0.94 (0.66-1.34)	0.82 (0.54-1.23)	1.26 (0.81-1.96)	1.71 (1.19-2.47)**	2.21(1.45-3.38)***	0.55 (0.16-1.86)
Health insurance at baseline							
No	1	1	1	1	1	1	1
Yes	0.79(0.50-1.27)	1.29 (0.79-2.09)	1.57 (0.92-2.69)	0.87 (0.49-1.55)	1.10 (0.67-1.82)	1.42(0.77-2.61)	0.95 (0.20-4.46)
Demographic changes							
Changes of residence							
Stable	1	1	1	1	1	1	1
Changed	1.49(0.83-2.68)	0.96 (0.52-1.75)	0.72 (0.36-1.44)	0.63 (0.32-1.24)	1.31 (0.72-2.40)	1.98 (0.94-4.18)	5.78 (1.28-26.04)*
Changes of health insurance							
Stable	1	1	1	1	1	1	1
From no to yes	0.91(0.61-1.36)	1.37 (0.90-2.08)	1.56 (0.98-2.47)	1.44 (0.85-2.45)	1.31 (0.85-1.99)	0.96 (0.60-1.54)	0.61 (0.13-2.83)
From yes to no	2.03(1.01-4.07)*	1.11 (0.54-2.27)	0.65 (0.29-1.45)	2.05 (0.76-5.50)	0.67 (0.31-1.44)	0.82 (0.34-1.98)	1.66 (0.23-12.05)
Health-Related Behavior and Changes							
Smoking at baseline							

No	1	1	1	1	1	1	1
Yes	1.01(0.52-1.96)	0.86 (0.44-1.68)	1.58 (0.72-3.45)	1.09 (0.47-2.51)	0.57 (0,27-1.16)	0.74 (0.36-1.51)	0.78 (0.03-17.67)
Smoking changes							
Stable & started smoking	1	1	1	1	1	1	1
Quitting smoking	0.95(0.48-1.88)	1.93 (0.89-4.17)	0.94 (0.40-2.20)	1.09 (0.45-2.59)	0.45 (0.18-1.06)	1.22 (0.55-2.73)	4.46 (0.93-21.36)
Physical Activity at baseline							
Low	1	1	1	1	1	1	1
Medium	1.89(0.71-5.02)	0.88 (0.33-2.31)	0.67 (0.18-2.42)	0.96 (0.28-3.24)	0.82 (0.29-2.31)	2.29 (0.75-6.95)	0.93 (0.01-58.21)
High	1.22(0.57-2.62)	0.74 (0.34-1.61)	1.01 (0.39-2.60)	0.83 (0.33-2.12)	1.09 (0.50-2.40)	1.12 (0.45-2.27)	2.83 (0.15-53.02)
Physical activity changes							
Stable	1	1	1	1	1	1	1
Reduced	0.95(0.50-1.81)	1.02 (0.52-1.99)	1.39 (0.64-3.01)	1.37 (0.62-3.02)	0.64 (0.32-1.25)	1.42 (0.63-3.17)	2.44 (0.24-24.67)
Increased	1.47(0.99-2.17)	1.20 (0.80-1.81)	1.81 (1.14-2.86)	1.29 (0.77-2.15)	1.17 (0.77-1.77)	1.81 (1.13-2.89)	2.80 (0.53-14.76)
Protein intake changes							
Low stable	1	1	1	1	1	1	1
Low to high	0.82(0.46-1.46)	0.45 (0.25-0.82)**	0.61 (0.30-1.26)	1.05 (0.48-2.30)	1.16 (0.61-2.18)	1.45 (0.71-2.95)	0.25 (0.02-2.83)
High to low	1.50(0.85-2.64)	0.88 (0.49-1.58)	1.21 (0.60-2.42)	1.13 (0.54-2.36)	1.16 (0.64-2.10)	1.40 (0.73-2.69)	2.43 (0.23-25.22)
High stable	0.98(0.56-1.72)	0.78 (0.43-1.40)	1.13 (0.56-2.27)	0.76 (0.37-1.56)	1.24 (0.69-2.26)	1.63 (0.84-3.15)	3.73 (0.43-32.35)
Age (70+)*Smoking(no)	1	1	1	1	1	1	1
Age (70+)*Smoking(yes)	1.79(0.52-6.12)	1.34 (0.41-4.33)	0.20 (0.02-2.01)	0.78 (0.16-3.76)	3.87 (0.98-15.16)	1.85(0.46-7.32)	2.40 (0.00-0.00)
Age(70+)*PA(low)	1	1	1	1	1	1	1
Age(70+)*PA(medium)	0.46(0.08-2.53)	1.44 (0.27-7.49)	1.50 (0.12-18.26)	410167136.70(0.00-0.00)	1.32 (0.17-9.91)	0.53 (0.07-3.79)	0.88 (0.00-0.00)
Age(70+)*PA(high)	1.11(0.35-3.48)	0.96 (0.32-2.91)	0.38 (0.06-2.31)	1.21 (0.26-5.52)	1.03 (0.22-4.70)	0.51 (0.13-1.89)	0.31 (0.00-0.00)
Age(70+)*Protein (low)	1	1	1	1	1	1	1
Age(70+)*Protein (high)	2.17(0.83-5.62)	0.69 (0.27-1.78)	2.96(0.74-11.87)	1.18 (0.33-4.18)	0.72 (0.20-2.58)	0.77 (0.26-2.30)	2.11 (0.00-0.00)
Education*Smoking(no)	1	1	1	1	1	1	1
Education*Smoking(yes)	0.60(0.26-1.38)	1.13 (0.52-2.44)	0.56 (0.24-1.31)	0.82 (0.33-2.03)	1.02 (0.45-2.25)	0.71 (0.29-1.73)	1.00 (0.09-10.76)
Education*PA(low)	1	1	1	1	1	1	1
Education*PA(medium)	1.24(0.42-3.68)	1.12 (0.39-3.23)	1.67 (0.45-6.12)	0.53 (0.14-1.97)	1.21 (0.38-3.90)	0.59 (0.16-2.09)	1.90 (0.06-57.31)
Education*PA(high)	1.24(0.83-1.85)	1.48 (0.96-2.28)	1.31 (0.73-2.33)	0.85 (0.51-1.40)	1.48 (0.98-2.24)	1.49 (0.70-3.16)	1.12 (0.41-3.07)
Education*Protein(low)	1	1	1	1	1	1	1
Education*Protein(high)	1.02(0.67-1.55)	1.34 (0.86-2.10)	1.00 (0.59-1.72)	0.93 (0.53-1.61)	1.10 (0.72-1.69)	1.37 (0.71-2.62)	3.43 (1.06-11.03)*

Note: Binary logistic regression was used for analysis. The reference group of the variables: chronic disease (have chronic disease), physical function (have physical function), depressive symptoms (have depressive symptoms), cognitive (have impaired cognitive function), social support (no having), social participation (no having), overall successful aging (failed), age (age 60-69), gender (women), monthly expenditure, residence (urban), ethnicity (Javanese), health insurance (yes), smoking (no), protein intake & changes (low stable), physical activity (low), changes of health insurance (stable), changes of residence (stable), physical activity changes (stable), smoking changes (stable & starting smoking). *$p<0.05$, **$p<0.01$, ***$p<0.001$.
